# Current Landscape of Psychologists in Academic Health Centers: Roles and Structural Models

**DOI:** 10.1007/s10880-024-10040-6

**Published:** 2024-08-23

**Authors:** Michelle B. Moore, Kelly Gilrain, Cheryl Brosig, Jarrod M. Leffler, Shikha Gupta, Philip Fizur

**Affiliations:** 1 Department of Psychiatry, Louisiana State University Health Sciences Center, 2021 Perdido Street, New Orleans, LA 70112 USA; 2 Department of Medicine, Cooper University Health Care, One Cooper Plaza, D227, Camden, NJ 08103 USA; 3 Department of Pediatrics, Medical College of Wisconsin/Children’s Wisconsin, 9000 W. Wisconsin Avenue, MS713, Milwaukee, WI 53201 USA; 4 Department of Psychiatry, Virginia Commonwealth University & Children’s Hospital of Richmond, 1308 Sherwood Avenue, Richmond, VA 23220 USA; 5 Department of Psychiatry, University of Missouri School of Medicine, 1 Hospital Drive, Columbia, MO 65203 USA; 6 Department of Medicine, Cooper University Health Care, One Cooper Plaza, Dorrance 253, Camden, NJ 08103 USA

**Keywords:** Psychologist, Roles, Models, Recruitment

## Abstract

With mental health needs consistently increasing in our communities and medical centers, we want to ensure that institutions are aware of the benefit and value that psychologists bring to their system and provide several pathways for consideration and structure to understand how to support the salaries and careers of psychologists working within AHCs. Leadership and administration within Academic Health Centers (AHC) often do not understand the value and measurement of productivity for psychologists being added to the medical teams. The current article aims to present varied structural models and demonstrate how productivity is reviewed for psychologists across different institutions and departments. The authors will outline the many roles that psychologists serve within academic health centers as well as the value those roles bring to the system. The overarching goal is to provide an educational article that serves as a tool for recruitment of psychologists that leaders and faculty can refer to when approaching administration in AHCs to understand the systems and roles of psychologists within medical settings. This information can be utilized to help create new positions for psychologists, aid in recruitment efforts and provide transparency for faculty currently working within AHCs who may not be aware of the varied opportunities.

## Introduction

Psychologists have been working within medical schools and academic health centers (AHCs) for over one hundred years (Robiner et al., 2014), and the numbers continue to grow. Despite this growing trend, they still represent a small portion of all medical school faculty and leadership roles within AHCs (LaPaglia et al., 2017). Factors for this may include administrators’ concerns about funding positions for psychologists, lack of awareness about the skill sets of psychologists other than clinical, and lack of representation and models for psychologists in various roles in AHCs. As Hong and Robiner (2016) report on the state of psychologists, they are integral to the academic mission of AHCs with their ability to integrate as scientists, educators and clinicians. With the changing landscape of healthcare to include greater interdisciplinary care, collaboration, and value-based care, psychologists are well-positioned to serve greater roles within these systems beyond their traditional roles (Kirch & Ast, 2017).

As early career faculty, most psychologists are recruited to primarily clinical or research positions. Given psychologists’ educational training in human behavior, they are often well-suited for positions related to wellness, mentorship, faculty development and leadership as they move forward in their career. As one advances across the faculty lifecycle described by Smith and Bunton (2012), they may experience shifts in their roles and responsibilities which create opportunities for advancement. When leadership uses a faculty lifecycle model, they can improve rates of retention and engagement throughout one’s career. While all phases of the cycle present with unique challenges, it is important to maintain awareness of the lifecycle even throughout the middle-phase of one’s career, which is often overlooked (Baldwin et al., 2005). Overtime, the psychologist’s role may evolve as leadership becomes more aware of the individual’s abilities to support other initiatives outside of the traditional clinical and research-based roles. As systems change and adapt, psychologists may also find increased opportunities for growth in addressing larger systemic challenges or advances that can have a greater impact on the institution and community at large.

Across the United States, a psychologist’s role within an AHC can look quite different between organizations. Administrators are often perplexed about where psychologists fall alongside physician colleagues and other health care professionals in terms of salary, work relative value units (wRVU) expectations and productivity. As institutions aim to build or grow psychology services, administrators may question what structural model works best for their setting and current institutional needs. The aim of this article is to provide descriptions of structural models that depict psychologists’ roles and responsibilities within AHCs. Throughout this article, we are referring to health service psychologists who primarily provide clinical services rather than those employed on a research track. When approaching administration and leadership to either develop a new psychology service, enhance existing services or consider changing the structure of a current program, this article can serve as an educational tool and resource to provide a blueprint for psychologist positions and programs. We illustrate three different structural models where psychologists may work within AHCs, which includes centralized, decentralized and integrated behavioral health. Within the same AHC, psychologists may be employed in more than one model that is described, and systems may be setup with a combination of multiple structural models. We aim to provide a guide that leaders and faculty can use when approaching administration in AHCs that offers a concrete understanding of the various systems and roles of psychologists, who provide primarily clinical services, within academic medical settings. An overview is also provided of the common expectations that exist for psychologists as clinicians and clinical educators working within AHCs regardless of the specific structural model in which they work. Lastly, psychologists can use this resource if they aspire to work within an AHC, are interested to learn about how other systems operate outside of their home institution or seek to further their professional advancement within AHCs through different roles and responsibilities that may be available to them.

## Structural Models for Organizing Health Service Psychologists Within AHCs

### Centralized Psychology Model in Psychiatry and Pediatrics Departments

At many institutions, the majority of psychologists are centralized within one department. At times, AHCs may have a freestanding Psychology department which offers the advantages of better workforce and workflow planning, a more consistent approach to productivity expectations, an increased sense of professional identity for psychologists, and enhanced mentoring to support psychologists as they work toward academic promotion (Rozensky, 2004). However, freestanding Psychology departments are often not feasible unless the medical school is part of a larger health sciences university which also offers graduate degrees in psychology. More commonly, centralized models for psychologists occur within departments of Psychiatry or Pediatrics. In these departments, psychologists often report to a division head or section chief for their work in that department; this person may or may not be a psychologist. Benefits of a centralized model in a department other than psychology can include more immediate access to colleagues of other disciplines for research, education and clinical activities, cross-pollination of ideas in established multi-disciplinary networking and meeting structure, consideration to engage in professional activities outside of traditional psychology work, and more readily available training opportunities for a range of learners. Possible disadvantages of a centralized model outside of a freestanding psychology department include department administrators having limited understanding of what psychologists can do and how their training and skillset differs from physicians.

Within many centralized department models, psychologists spend the majority of their time in clinical care and teaching and are typically on the Clinician-Educator track for promotion. However, faculty are expected to pursue scholarly activities, which are outlined later in the article. Faculty full-time equivalent (FTE) is often broken down by percent of effort that is devoted to each of the academic missions (clinical, teaching, research, etc.). Clinical productivity metrics vary by institution and may be based on a set number of hours spent in face to face clinical care, or based on specialty wRVU benchmarks for psychology. Examples of wRVU benchmarks that are commonly used include the Medical Group Management Association (MGMA) and the Association of Administrators in Academic Pediatrics (AAAP). As noted by Dawson & Speelman, 2023), there are potential disadvantages to measuring productivity with wRVUs alone due to differences between specialty areas, the type of clinical practice (outpatient, inpatient or a combination of the two), and the billing codes that are used (Health and Behavior Assessment and Intervention vs. mental health CPT codes). In addition, wRVUs do not fully capture the non-billable hours that are required for quality patient care. Of note, clinical productivity metrics may be reduced if a psychologist has a significant teaching role or receives a grant that funds part of their salary. It is important for psychologists to understand the expectations for their faculty role, what percent of their time will be spent in the various academic missions, and which clinical productivity metric is being used by the institution, particularly when considering a new position.

### Decentralized Psychology Model

At some AHC’s, there is a decentralized psychology model, and psychologists are hired by multiple departments and report to their respective clinical leaders, who are often physicians. Some departments may include Medicine, Psychiatry, Neurology, Infectious Disease, Pediatrics and Addiction Medicine. For example, in one de-centralized model, there is one lead Chief Psychologist who is responsible administratively for all of the psychologists across the healthcare system. Creating a clinical department within the medical staffing office will allow for the Chief to support with credentialing, reviewing Focused Professional Evaluations and Ongoing Professional Evaluations to ensure clinical alignment with privilege specific competency. This lead role typically coordinates with the Chief Physician Executive and supports all psychologists in understanding and ensuring productivity requirements, creating incentive plans, working with finance on market analysis, ensures that the team is fairly compensated, and ensuring any challenges related to contracts, compensation, roles, and expansion of responsibilities across the healthcare system are fairly addressed for all psychologists. One way to create an incentive plan is the creation of scorecards that take into account finance, access, quality, service, growth and academic contributions to the healthcare system. In this model, the Chief would work with each department to think through how to schedule patients to maximize productivity. Having a psychologist as lead in this area is beneficial as we have more of an understanding of which codes might best be used in varying structures and can advocate as needed to modify expectations, rather than leaving this to a medical colleague. More often than not, using standard psycho-diagnostic exam and psychotherapy codes, psychologists can reach their targets. Moreover, having a psychologist as lead in this role, allows for thoughtful consideration to how appointments are scheduled (i.e.,—30 versus 60-min settings, weekly or bi-monthly) as it relates to psychological clinical care. For instance, there is a great deal of collaboration in many interdisciplinary clinics, and having a psychologist chief, who supports and makes recommendations across all areas, allows consideration and flexibility for scheduling so that psychologists can reach targeted goals by maintaining 6 or more (45–60 min sessions) to 10 (30 min sessions) patients scheduled per day. also If working in a primary care or specialty ambulatory clinic there is also opportunity to obtain warm handoffs from providers during their medical visits, which is supportive particularly if there are any no-shows for scheduled sessions. For inpatient CL psychologists, productivity may fluctuate, however, with support from one’s chief, identifying logistics to secure consults, triaging areas with high users of psychological supports, while understanding the cognitive load it takes to provide assessment and intervention for numerous patients per day, allows for additional support of ensuring these teams reach goals. Similarly, neuropsychologists, have room for a varied approach and combination of intake interviews, neuropsychological assessments, and feedback sessions. Scheduling one to two (if the battery is shorter) patients per day or stacking four or more clinical interviews or feedback sessions per day, allows a neuropsychologist an opportunity to reach their clinical goals. Acknowledging this, it is worth considering what this looks like practically in the daily life of psychology faculty at a given AHC.

Beyond RVUs, billing and collections is also an important aspect to consider regardless of whether a centralized or decentralized model is used. Collection of billing will vary from institution to institution and state to state. It is important that psychologists have ongoing conversations with their billing teams to ensure that contracts with payors are up to date and there are no barriers with collecting payment for psychologists’ work. Regular reviews of denial payments by billing teams will help to target if there are any challenges with collections for certain payors. One last thought to consider when discussing structures with leadership is to present the ‘soft dollars’ that occur by having psychologists within one’s institution. This may be the time saved for physicians as they can see a larger quantity of medical patients by having psychologists able to address the mental health issues side by side. This might also be seen in reducing costs on throughput by facilitating discharges. Lastly, it may be that by adding psychologists to a medical team which leads to supporting providers and staff emotionally, engagement is improved, and turnover rates may be lowered, which both save costs to the healthcare system.

Choosing the correct model is a larger conversation to be had specific to institutions. Finance teams and those who support mental health care. There is no one right or wrong answer, simply what fits best within the system.

### Integrated Behavioral Health Model

Having outlined the administrative evolution of psychologists in academic medicine it is worth considering how the field's clinical organization continues to grow, which is highlighted well by the proliferation of the Primary Care Behavioral Health (PCBH) model. Per Robinson and Reiter (2016)​, this model emphasizes the responsibilities of identifying, treating, and triaging patients; integrating psychological principles in education and care in medical setting to help manage increasingly common medically and behaviorally complex presentations; serving as consultants to all other professions; educating on and promoting population-based care philosophies; and enhancing behavioral health services to assure consistent and seamless collaborations between primary care and specialty services. This is achieved through the GATHER method (Reiter et al., 2018). First, being a Generalist who are Accessible to the team at all times through warm hand-offs and electronic referrals, working with patients of any age and any behavioral concern. This means promoting a Team-based approach, participating in meetings and huddles about patient care, with all of the above leading to High productivity, seeing a large number of patients each day. Behavioral health consultants (BHC) should be Educators, synthesizing interdisciplinary information to help patients and colleagues better understand health and behavior in the psychosocial context. Finally, the BHC creates Routine for normalizing interdisciplinary referrals in the practices’ workflow, and in doing so normalizing and destigmatizing behavioral health care (Reiter et al., 2018).

But what does this look like practically speaking for a psychologist at present? This depends on the institution. For example, an AHC utilizing the PCBH model in both primary and specialty care clinics can expand the clinical services available beyond the traditional clinical interventions. These clinical services may include brief yet comprehensive neuropsychological assessment, as well as incorporating all levels of mental health services from social work to psychiatry, and across all levels of training. In this model, psychology is at the center, which highlights for the hospital community broadly and leadership specifically the invaluable nature of the psychologist as clinicians and leaders within the clinic teams.

Using the most recent data available (2021), psychologists working in a PCBH model exceed the MGMA Median RVU by 11% on average across tracked clinics. With recent changes to available billing codes, both expanding existing codes and adding new codes, these numbers are estimated to increase. These code changes highlight the evolving role of psychologists in AHCs and include, but are not limited to, Group Caregiver Behavior Management Training CPT® Code 96,202; Interprofessional Telephone/Internet/Electronic Health Records Consultation 99,446–99,449; “Incident to” billing; and G0323 for General Behavioral Health Integration. Anecdotal data gathered from clinics that more recently began to implement this model, including women’s health, other primary care offices, and pediatrics, suggest these trends will hold.

Building on this, there is reason to believe this model will become increasingly effective in the coming years as master’s level and other similar providers join the behavioral health team and as their professions continue to advocate for such involvement (Tadic et al., 2020). Increasingly, social workers are expected to practice to the top of their licenses, and many are equipped to practice independently in ways that overlap with what psychology offers in the PCBH (APA, 2023). However, both professions also have unique skillsets and, when focusing on those unique aspects, can enhance the primary care experience (Falender, 2018). More to the point, social workers can increasingly bill for behavioral interventions independently however, for those who cannot, psychologists can serve in a supervising capacity and bill the previously mentioned incident-to billing. However, when social work is billing independently, this allows psychologist to employee their unique skills. These include, but are not limited to, educating other members of the team, conducting research to improve care and refine the PCBH as well as aiding physicians in their own research design and implementation, measure development, quality improvement, as well as engaging in institutional, local, and national legislative work, and beyond (Dobmeyer et al., 2016).

Psychologists in the PCBH model have continued to increase their impact beyond the services noted above. Increasingly, a flexible approach to neurocognitive assessment in non-neurological clinics has expanded. Whereas highly complex neuropsychological consults continue to be appropriately referred to neuropsychologists in neurology, flexible batteries are increasingly being implemented to answer less complex referral questions, adding to the richness of information available to other team’s members in primary and specialty care settings and closing the established gap in access to these services.

Finally, psychologists in this model have increased their value not only through direct care but medical education as well. Using the principles of Interprofessional Education (IPE), psychologists have demonstrated their unique contribution to education in their own field as well as other professionals present in our system from undergraduate and graduate medical education, social work, nursing, and pharmacy (Robiner et al., 2014).

As outlined by Robiner et al. (2014), the increasing receptiveness of patients and physicians to psychological insights and processes in healthcare is producing better outcomes in both educational attainment and clinical care. This is important, as Robiner points out, given psychology’s expertise in learning, group processes, motivation, and the inherent interplay of biopsychosocial factors in health outcomes. As IPE efforts have increased, psychologists have increasingly been involved in roles including didactic trainings, medical rounds, clinical precepting, direct supervision, mentoring, and facilitating research and other educational projects with faculty members and learners within and across disciplines.

### Expectations of Psychologists in AHCs Across All Models

Outside of psychologists’ clinical responsibilities are those activities, (i.e., teaching, research, scholarly activity, service to the institution and administration), which align with the academic mission and support a faculty when considering academic promotion and advancement. In the area of teaching, psychologists may focus efforts in many areas across the institution. For many models, departments are responsible for teaching and supervising psychology practicum students, interns, and post-doctoral fellows as part of APPIC accredited programs. Programs that are accredited must adhere to specific standards to maintain accreditation, which is a large responsibility on the faculty. Faculty engage in teaching and mentoring other learners within, or across, departments which could include psychiatry, pediatric, neurology, medicine residents and fellows. Outside of the department level, faculty may be involved in undergraduate medical education courses primarily as course instructors and facilitators in lectures and active learning sessions as well as serving as facilitators for interprofessional education sessions as part of the health sciences university.

In the area of research and scholarly activity, psychologists are fortunate to collaborate with basic science researchers from other departments in the health sciences center who need behavioral health consultants as part of their research. These opportunities can be funded and support part of the psychologists’ salary by buying out clinical time. Faculty research projects typically involve medical students and other trainees which allows for mentorship in scholarly activities, and faculty are also active in presenting at local, regional, and national conferences on topics within their area of clinical expertise. As mentioned, many faculty members are hired on a clinical track; therefore, most faculty do not rely on research funding to support their salary. Research conducted by psychologists typically relates to their area of clinical specialty and is funded by internal pilot grant mechanisms or philanthropy. Depending on the institution, extramural funding may or may not be required. Faculty who are interested in primarily serving as researchers are often hired on a traditional path for promotion and have established metrics for securing extramural funding after a certain period of time in order to partially support their salary. Faculty across all models are expected to engage in activities such as quality improvement, research, presentation at national meetings, and publication of their work in peer-reviewed journals.

In the administrative and service area, psychology faculty are engaged in several directorships within hospitals and medical schools. In addition to overseeing psychology training programs, psychologists also hold leadership roles for wellness and faculty development, trauma-informed care, and diversity, equity and inclusion. It is notable that psychology faculty are often appointed to a number of educational committees, assuring that the contributions of psychology as a field are well integrated into every aspect of undergraduate and graduate medical education. They address multiple content areas fundamental to medicine. Their participation in medical education and professional development activities for faculty are well-regarded. As healthcare becomes more interprofessional, with services delivered via interprofessional teams, opportunities for psychologists to contribute to, and play leadership roles in, IPE are expanding. It is critical that psychologists seize them.

## Additional Roles for Psychologists

Shaffer et al. (2021) reported on a survey completed by members of the Association of Psychologists in Academic Health Centers (APAHC) in 2017 to understand current leadership roles held by psychologists in AHCs. Of the 105 respondents to the survey, 68% of the respondents held leadership positions within their institution. The leadership positions included training psychology learners (49.5%), clinical service (33.3%) and research (26.7%). At that time, 11.5% of respondents held leadership positions within their system’s administration. Given the unique skill set of psychologists, they are excellent candidates for administration and organizational leadership positions.

Some of the leadership positions held by psychologists in clinical service may include clinic directors, service line directors, division chiefs or chairs, specific school (e.g., school of medicine) roles, department vice chairs or chair positions, or deans. Other leadership positions have been in training of psychology trainees, research and on faculty committees. While these are the more traditional roles that psychologists have played and continue to have incredible value, additional roles for psychologists are worth examining as well.

Another area that psychologists can make significant contributions is in learner training in various departments. This certainly would include psychiatry residents, who benefit in their work by learning the skills and interventions that are used by psychologists. More specifically, psychologists’ unique skill set in psychotherapy and supervision enhances the training opportunities in other programs. Having a psychologist as program director or associate program director in psychiatry, family medicine, or other departments seeing significant mental health issues, creates a more robust training program.

One growing area in AHCs, and in large organizations as a whole, is workforce wellness. More healthcare institutions are employing a Chief Wellness Officer, or similar position, to address the physical, mental and emotional health of the system and its employees. A psychologist has many of the skills to be successful in this position, including skills that they used in their clinical work. To engage in workplace wellbeing successfully, an individual needs to listen to their system’s employees, understand and contextualize the issues that are contributing to a lack of wellbeing, and then implement interventions that are tailored at the individual and systemic level.

As we further understand the roles psychologists play, we must also consider how this may vary depending on the system. Understanding the system’s model is important in recognizing reporting structures, navigating the AHC, and determining factors to consider when building a program.

## Future Directions for Psychologists in Academic Health Centers

While the above outlines how models of psychology have evolved administratively and clinically, there are still more exciting changes possible for the field. Psychologists have been working in AHCs in various roles involving clinical care, teaching, and research, as described in this article. Psychologists have been increasingly engaged in more administrative and leadership positions as the landscape of AHCs evolves. AHCs have been focusing more on integrated and collaborative care, which calls upon the skills that most psychologists have by training. Skills such as relationship building, emotional intelligence, effective communication, and systems improvement are often part of a psychology training program and are particularly useful in becoming a successful leader. These elements are critical to day-to-day services, and now that US News and World Report (USNWR) is providing rankings on behavioral health, the activities psychologists engage in will be reflected in organizations’ national rankings. For example, Neonatology, Nephrology, Gastroenterology & GI Surgery, and Diabetes & Endocrinology, receive additional points in their rankings when there is a psychologist on their service (Olmsted et al., 2023). As the USNWR rankings evolve they will include items more focused on behavioral health providers and programs with specific recognition of psychologists within these areas.

Shaffer et al. (2021) outline the many qualities that psychologists possess to make them successful leaders in AHCs as well as areas that psychologists may still need to develop. These include skills in financial and personnel management, program development, and human resources. Creating programs for leadership development for psychologists would be mutually beneficial for psychologists as well as the institutions in which they work.

The authors of this article encourage psychologists to continue to grow professionally by enhancing their clinical skills and research experience as well as utilizing and expanding their leadership skillset. This may be done by attending trainings held for psychologists in or desiring leadership positions, professional mentorship, or leadership coaching. Utilizing these resources sets psychologists up for further advancement into more complex roles in AHCs that may require skills such as budgeting and financial forecasting, team communication, quality and process improvement, and public speaking.

A successful leader requires having team leadership skills, effective communication and interpersonal skills, time and task management skills, and much more. These are skills that can be learned and practiced with a growth mindset and continued learning throughout one’s career. It will benefit psychologists to move beyond the clinical skills they already know and explore other areas of development.

One of the major benefits of working within an AHC compared to private practice is the diverse opportunities for psychologists in terms of their job duties and positions. With the growing demand for behavioral health services in healthcare as well as a desire to expand medical education to incorporate more behavioral health, now is a ripe opportunity for psychologists to explore and advance their careers in AHCs. The various types of structural models laid out in this manuscript, as well as logistics of funding psychologists in various roles, can be used as a resource for psychologists navigating administration in their institution. Psychologists are encouraged to expand upon their current skillset to further sharpen skills valuable for leadership positions as we believe that this will be beneficial for psychologists in their careers as well as for academic healthcare institutions.

## Data Availability

Not applicable.
